# The Attitudes of Relatives of ICU Patients toward Informed Consent for Clinical Research

**DOI:** 10.1155/2020/2760168

**Published:** 2020-10-09

**Authors:** Rania Mahafzah, Karem H. Alzoubi, Omar F. Khabour

**Affiliations:** ^1^Dept. of Clinical Pharmacy, Jordan University of Science and Technology, Irbid 22110, Jordan; ^2^Dept. of Medical Laboratory Sciences, Jordan University of Science and Technology, Irbid 22110, Jordan

## Abstract

**Background:**

Informed consent is a key ethical requirement for biomedical research that is implemented to ensure autonomy and voluntary participation. However, patients in the intensive care unit (ICU) may be unconscious or severely ill and thus lack the capacity for decisions about research participation. Thus, relatives or guardians are usually asked to provide informed consent prior to the inclusion of ICU patients in research.

**Aims:**

This study aimed to assess the attitudes and preferences of relatives of ICU patients toward informed consent in biomedical research in Jordan. *Subjects and Methods.* A sample of 184 relatives with a critically ill next of kin in the ICU was anonymously surveyed regarding their attitudes and preferences toward giving informed consent for biomedical research on behalf of their patients.

**Results:**

The study showed that the majority of relatives had a positive attitude toward the informed consent process on behalf of their patients in the ICU (72.3%). The perception that participation in research would be directly beneficial to their patient was the most significant reason to provide informed consent among relatives. The degree of relatedness to the patient was significantly associated with the decision to provide informed consent on behalf of the patients in the ICU. Additionally, more than 70% of the relatives strongly agreed to take part in clinical research if they were to be unconscious patients in the ICU. Moreover, the majority of the respondents agreed that their first-degree relatives would give consent on their behalf.

**Conclusion:**

Relatives with a critically ill next of kin in the ICU had positive attitudes toward providing informed consent on behalf of their patients. This was motivated by the direct benefit from the research to their patient.

## 1. Introduction

Clinical research in the intensive care unit (ICU) is essential to improve patients' care and quality of life [1]. A central requirement for research approval from institutional review boards (IRBs) is informed consent to be obtained from subjects before participating in research. This requirement is reflected in the Belmont report under the “respect of person” ethical principle, where human subjects should be treated as autonomous persons who provide consent for research without being coerced or deceived [2, 3]. However, most of the patients in the ICU are considered vulnerable and entitled to additional protection, as their mental status has been altered ranging from confusion, unconsciousness to coma, which prevents them from making decisions to participate in research [4, 5]. The proxies are the most frequent alternative for making the substituted judgment about participating in biomedical research on behalf of patients in the ICU [6]. This substituted judgment needs close knowledge about patients' values and preferences, which applies only to close family members [6]. In many countries, the relatives are recognized as an alternative legal representative for informed consent decision on behalf of ICU patients [7–9]. The consent approval rate by relatives of the patients in the ICU was shown to vary from about 50% to 90% [10]. However, researchers should ensure that relatives have the mental capacity to consent in the context of the ICU settings, thus be able to provide a “genuine proxy consent” [6]. To accommodate for that, various criteria should be utilized to obtain accurate consent form for research participation of patients in the ICU [5, 11, 12]. The accurate substituted judgments should be morally valid, reflecting the beliefs of the patients and respecting the autonomy of the patients [3, 6]. Therefore, obtaining informed consent from the patients in the ICU is a challenge and could pose an ethical dilemma for the researchers [7, 13].

The attitudes and preferences of relatives of ICU patients toward informed consent for clinical research were not widely investigated [14, 15]. Based on our knowledge, there has been no formal study of proxy consent of ICU patients in Jordan. Therefore, the objective of the current study was to investigate the general attitudes and preferences of relatives toward informed consent for biomedical research on behalf of their unconscious patients in the ICU.

## 2. Subjects and Methods

### 2.1. Participants and Procedure

A total of 200 ICU patient's relatives (≥18 years) were invited to participate in this cross-sectional survey study. The nearest relatives of ICU patients were included such as parents, spouses, sons, or daughters. The sample of the study was obtained from visitors in the waiting rooms of the two ICU units at King Abdulla University Hospital in the north of Jordan during the period of March-June, 2019. Both of the ICU units at KAUH are general ICUs with a capacity of 120 beds. These ICU units have a number of clinical trials ongoing. Participants who were invited were asked to complete an anonymous paper-based questionnaire designed to assess relatives' attitudes toward giving informed consent for research on behalf of their patients in the ICU. Before participation, potential subjects received a full description of the study by a trained researcher, who was also a clinical pharmacist at the ICU unit. Written informed consent was obtained from all study participants.

### 2.2. Questionnaire Construction

A structured questionnaire was developed to evaluate the attitudes of relatives of ICU patients toward providing informed consent for research studies. To measure the validity of the questions, three experts in this field who included a researcher, a physician, and an ethicist reviewed and tested the validity and readability of the content of the survey to ensure that items are appropriate for assessment. Additionally, pilot testing on 20 participants was done to ensure clarity and comprehension of the questions. The questions were in the Arabic language and could be completed in 5 minutes based on the pilot test.

The questionnaire provided a brief explanation about severe illness-related decisional impairment among ICU patients. Along with the need to conduct biomedical research, the research requires informed consent from the relatives on behalf of their patients as they are considered legally authorized representatives in accordance with local regulations and national laws in Jordan. The survey briefly explained few basic phenomena related to biomedical research, e.g., patients who participate in a study will receive therapeutic and/or nontherapeutic practices that could drive direct benefits as well as potential risks to the patient. However, future patients and society at large will benefit from the knowledge that is to be gained from research studies. The study questionnaire also provided a definition of proxy consent and dual consent. The participants were provided with contact numbers of the principal researcher and the human research ethics committee officer in case they opted to provide any concerns regarding the questionnaire.

The survey covered three domains. The first domain collected information regarding demographics of the study participants such as age, gender, level of education, and relationship to the patient. The second domain assessed the responses of relatives of ICU patients toward giving informed consent for research. To establish their beliefs about biomedical research, the participants were asked if they would agree to participate for direct benefits to their patients or for supporting the research to optimize future treatments. In the third domain of the questionnaire, the participants were asked if they were to be placed in a critical disabled situation from making an informed decision, would they agree to consider their relatives to provide informed consent on their behalf. The questionnaire items were in multiple-choice format, and each participant was allowed to choose only one choice as a response.

### 2.3. Statistical Analysis

Statistical analysis was conducted using SPSS, version 21. Demographic data and categorical variables were summarized using frequency tables. Chi-squared test was used to assess associations among variables. A *P* value of <0.05 was considered statistically significant.

## 3. Results

### 3.1. Demographic Characteristics

A total of 184 of the 200 ICU patient's relatives approached filled the questionnaire giving a response rate of 92%. The results revealed that 35.9% of the respondents had one of their parents in the ICU, while 15.2%, 20.1%, and 28.8% of respondents had a spouse, a sibling, or an offspring, respectively, admitted to the ICU. The gender distribution was 55.4% males and 44.6% females. The majority of participants were 40 years old or older, had a university degree, and were employed (Table 1).

Table 2 shows relatives' attitudes toward informed consent on behalf of their ICU patients for the purpose of participation in a clinical study. Of the respondents, 72.3% strongly agreed to provide informed consent on behalf of their patients in the ICU. The reason behind this positive attitude was mainly perceiving research study participation as being beneficial to their patients in the ICU (69%). Only 13.0% of the relatives of ICU patients indicated that their positive attitude to provide informed consent was only to support scientific research. Age, educational level, and gender did not impact attitude toward participation (*P* > 0.05, Table 3). However, relatedness to the patient was significantly associated with the decision to provide informed consent on behalf of the ICU patients (*P* < 0.05) and the perception of additional benefits to their patients (*P* < 0.05).

The relatives were also asked about their attitude towards participation in clinical research if they were to be an unconscious patient in the ICU (Table 4). More than 70% of the participants strongly agreed to take part in clinical research if they were to be unconscious patients in the ICU. Furthermore, in response to the question of “Who can provide informed consent for research participation on your behalf?” 57.1% of the participants answered that their first-degree relatives should give the consent, whereas 15% stated that the treating physician or the ICU consultant should give the consent (Table 4). Still, 7.1% believed that informed consent is not necessary. Demographic variables were not associated with any of the after-mentioned attitudes of patients' relatives (Table 5).

## 4. Discussion

This survey was performed in response to the lack of available community data about the attitudes and preferences of relatives toward making a surrogate decision on behalf of their patients in the ICU. Relatives' attitudes toward research and if relatives would agree to participate themselves in a biomedical research if placed in a critical situation that disable them to decide were investigated. The selected sample of the current study was from relatives who have gone through an experience of admission of a closely related patient to the ICU during the study period. Approximately 70% of the respondents indicated that they would agree to provide proxy consent on behalf of their patients in the ICU. The respondents were more likely to provide informed consent if the research was considered to be directly beneficial to their patients. Demographic factors such as age, gender, and education level did not have a significant effect on responses. However, the relatedness of the respondents with patients in the ICU significantly affected the rate of providing informed consent by the relatives on behalf of patients. The respondents who had father/mother and who had a son/daughter admitted to the ICU indicated that they would significantly have a positive response to provide informed consent on behalf of their patients for medical care procedures rather than for research.

Substituted judgment is common in critical care research, but the validity of ethical and legal issues should be evaluated. The correlation between patients and proxies is vital for obtaining a valid substituted judgment. Close family members have the ability to reflect the patients' preferences and views [6]. Current results have shown that respondents were mostly willing to provide informed consent for research associated with direct benefits to their patients rather than for the mere support of biomedical research. This finding highlights the possibility of proxies to be vulnerable to potential therapeutic misconception. Generally, most of the research participants are recruited from hospital settings by clinicians and healthcare providers where the participant may fail to distinguish between standard clinical practices and research practices. Additionally, in the ICU research settings, the proxy consent may be biased by the emotional burden to relatives for saving the life and decreasing the suffering of their beloved patient [16].

The IRBs and researchers should establish the shape of genuine proxy consent. The idea of consent has been discussed mainly as a legal decision to protect the researchers and medical institution [6]. Over the time, the informed consent has been developed to become a fundamental component of both the legal and ethical requirements of respect to persons/autonomy principle [9]. In Jordan, there is conformity with local legislations required for the medical procedures. Close family members are considered legally authorized representatives in accordance with the local medical laws in Jordan. Local IRBs allowed to use proxy consent on behalf of vulnerable patients such as patients with mental disorders, elderly, and children either for clinical practices or research practices. For ICU patients, substituted judgment is a common exercise by a close family member [2, 6]. However, for substituted judgment to be ethical, the proxies should have the capacity to consent after understanding three fundamental aspects: (1) voluntary participation, (2) benefits achieved from the research for both future patients and society, and (3) incremental nontherapeutic risks from the research [6].

Additionally, in terms of decision sharing or dual consent, the majority of participants indicated their ICU physician as the person to share the decision with. In consistence, a previous Canadian study indicated that most of the surrogate decision-makers preferred to share the decision with their healthcare provider [17]. It has been further demonstrated that relatives of patients preferred to share the decision with ICU physicians, hoping that this will help them in taking the decision in this acutely and stressful setting [3]. Dual consent was determined as a preferable model to overcome the mistrust of participants toward research [18]. On the other hand, dual consent may have more influence in shared decision-making, where the proxies could be biased toward physician decisions with the perception of providing their beloved patient with new treatment that is perceived to be better than the standard therapy [18]. To overcome this, an independent physician could be asked to make the dual consent with relatives and to provide suspected incremental risks compared with the clinical practices [19]. In Jordan, dual consent as an alternative to informed consent for patients in the ICU is not fully implemented. Accordingly, further studies are needed to investigate the effect of involvement of physicians in the informed consent process and how such involvement could be relieving the emotional burdens of proxies, in the face of the likelihood of introducing the possibility of therapeutic misconceptions in the view of the potential conflict of interest on the part of the treating physician.

The survey of the current study had a number of limitations including sampling only relatives from a single medical center, limited number of questions which may have led to response bias, and the use of quantitative survey which does not provide additional data about why participants provided certain responses or to explore their attitudes in further detail. Further, a comprehensive study is needed probably using more question options via an unstructured survey to better assess the complex perceptions and emotions of the relatives and measure the causes of agreement and disagreement with the informed consent process for research.

The current study represents the first step to discuss potential ethical challenges regarding proxy consent on behalf of their patients at an ICU setting in Jordan. The current study serves to alert researchers to potentially inaccurate surrogate decisions. However, involving patients in research is not a simple process. Patients have beliefs, preferences, and expectations which are affected by knowledge, trust, previous experiences, and social and economic factors [2]. Researchers must consider the mental capacity of patients during the informed consent process to ensure the protection of patients involved in the research studies.

In conclusion, the willingness of the relatives to provide consent on behalf of their patients in the ICU was the major finding in this study and reflected a positive attitude within this regard. Dual consent involving the physician was also accepted by the relatives. Finally, the degree of relatedness is a crucial factor that influences the basis upon which the informed consent decision is made.

## Figures and Tables

**Table 1:** Characteristics of ICU patient's relatives.

Demographic characteristics of relatives (*n* = 184)	*N* (%)
Gender	
Male	102 (55.4%)
Female	82 (44.6%)

Age	
18–30	24 (13.0%)
31–40	53 (28.8%)
41–50	55 (29.9%)
>50	52 (28.3%)

Education status	
Primary school	21 (11.4%)
Secondary school	40 (21.7%)
Diploma	38 (20.7%)
University degree	85 (46.2%)

Relatedness	
Spouses (husband/wife)	28 (15.2%)
Parents (father/mother)	66 (35.9%)
Children (son/dughter)	53 (28.8%)
Siblings (sister/brother)	37 (20.1%)

**Table 2:** Relatives' attitudes toward informed consent for clinical research on behalf of the patients in ICU.

Questions	*N* (%)
Do you agree to provide informed consent for participation in a clinical study on behalf of your patient in ICU?	
Agree	133 (72.3%)
Neutral	8 (04.3%)
Disagree	43 (23.4%)

I agree because I think that the study will be beneficial for my patient	
Agree	128 (69.6%)
Neutral	8 (04.3%)
Disagree	48 (26.1%)

I agree because I want to support the scientific research and optimize future therapeutic knowledge	
Agree	24 (13.0%)
Neutral	36 (19.6%)
Disagree	124 (67.4%)

**Table 3:** Attitudes of ICU patient's relatives toward informed consent according to their demographic factors.

Demographic factors	Do you agree to provide informed consent for participation in a clinical study on behalf of your patient in ICU?				I agree because I think that the study will be beneficial for my patient				I agree because I want to support the scientific research			
	Agree	Neutral	Disagree	*P* value	Agree	Neutral	Disagree	*P* value	Agree	Neutral	Disagree	*P* value
Gender				0.514				0.503				0.206
Male	72 (70.6%)	6 (5.9%)	24 (23.5%)		69 (67.6%)	6 (5.9%)	27 (26.5%)		17 (16.7%)	21 (20.6%)	64 (62.7%)	
Female	61 (74.4%)	2 (2.4%)	19 (23.2%)		59 (72.0%)	2 (2.4%)	21 (25.6%)		7 (8.5%)	15 (18.3%)	60 (73.2%)	

Age				0.491				0.521				0.069
18–30	20 (83.3%)	0 (0.0%)	4 (16.7%)		20 (83.3%)	0 (0.0%)	4 (16.7%)		2 (8.3%)	4 (16.7%)	18 (75.0%)	
31–40	39 (73.6%)	4 (7.5%)	10 (18.9%)		36 (67.9%)	4 (7.5%)	13 (24.5%)		2 (3.8%)	16 (30.2%)	35 (66.0%)	
41–50	36 (65.5%)	2 (3.6%)	17 (30.9%)		35 (63.6%)	2 (3.6%)	18 (32.5%)		9 (16.4%)	8 (14.5%)	38 (69.1%)	
>50	38 (73.1%)	2 (3.8%)	12 (23.1%)		37 (71.2%)	2 (3.8%)	13 (25.0%)		11 (21.2%)	8 (21.2%)	33 (63.5%)	

Education status				0.986				0.991				0.228
Primary	15 (71.4%)	1 (4.8%)	5 (23.8%)		14 (66.7%)	1 (4.8%)	6 (28.6%)		2 (9.5%)	4 (19.0%)	15 (71.4%)	
Secondary	31 (77.5%)	1 (2.5%)	8 (20.0%)		29 (72.5%)	1 (2.5%)	10 (25.0%)		5 (12.5%)	9 (22.5%)	26 (65.0%)	
Diploma	26 (68.4%)	2 (5.3%)	10 (26.1%)		25 (65.8%)	2 (5.3%)	11 (28.9%)		10 (26.3%)	6 (15.8%)	22 (57.9%)	
University	61 (71.8%)	4 (4.7%)	20 (23.5%)		60 (70.6%)	4 (4.7%)	21 (24.7%)		7 (8.2%)	17 (20.0%)	61 (71.8%)	

Relatedness				0.14				0.035				0.006
Husband/wife	17 (60.7%)	4 (14.3%)	7 (25.0%)		17 (60.7%)	4 (14.3%)	7 (25.0%)		3 (10.7%)	10 (35.7%)	15 (53.6%)	
Father/mother	56 (84.8%)	2 (3.0%)	8 (12.1%)		53 (80.3%)	2 (3.0%)	11 (16.7%)		4 (6.1%)	13 (19.7%)	49 (74.2%)	
Son/daughter	36 (67.9%)	1 (1.9%)	16 (30.2%)		35 (66.0%)	1 (1.9%)	17 (32.1%)		14 (26.4%)	8 (15.1%)	31 (58.5%)	
Sister/brother	24 (64.9%)	1 (2.7%)	12 (32.4%)		23 (62.2%)	1 (2.7%)	13 (35.1%)		3 (8.1%)	5 (13.5%)	29 (78.4%)	

**Table 4:** Attitude of the relatives toward participating in clinical research.

Questions	*N* (%)
Suppose you are unconscious patient in ICU: would you agree to participate in clinical study?	
Agree	142 (77.2%)
Neutral	1 (00.5%)
Disagree	41 (22.3%)

Who would you accept to provide informed consent on your behalf?	
One of first-degree relatives	105 (57.1%)
Physician or ICU consultant	28 (15.2%)
No informed consent necessary	13 (07.1%)

If the researcher wants to make dual consent, where another person will share the consent decision on your behalf with your relatives, who you prefer to share the decision with your relatives?	
Sharing with another member form my relatives (relatives-relatives consent)	70 (38.0%)
Sharing with physician (physician-relatives consent)	114 (62.0%)

**Table 5:** Attitudes of patient's relatives towards participating in clinical studies if they were unconscious patients in ICU per demographic variables.

Demographic variables	Suppose you are an unconscious patient in ICU:										
	Would you agree to participate in clinical study?				Who would you accept to provide informed consent on your behalf?				If the researcher want to make dual consent, where another person will share the consent decision on your behalf with your relatives, who you prefer to share the decision with your relatives?		
	Agree	Neutral	Disagree	*P* value	First-degree relatives	Doctor	Informed consent is not necessary	*P* value	Sharing with another member from my relatives	Sharing with physician	*P* value
Gender											
Male	76 (74.5%)	1 (1.0%)	25 (24.5%)	0.467	57 (55.9%)	15 (14.7%)	6 (5.9%)	0.690	39 (38.2%)	63 (37.8%)	0.538
Female	66 (80.5%)	0 (0.0%)	16 (19.5%)		48 (58.5%)	13 (15.9%)	7 (8.5%)		31 (61.8%)	51 (62.2%)	
Age											
18–30	21 (87.5%)	0 (0.0%)	3 (12.5%)	0.588	13 (54.2%)	3 (12.5%)	5 (20.8%)	0.107	10 (41.7%)	14 (58.3%)	0.934
31–40	41 (77.4%)	1 (1.9%)	11 (20.8%)		32 (60.4%)	11 (20.8%)	0 (0.0%)		19 (35.8%)	34 (64.2%)	
41–50	40 (72.7%)	0 (0.0%)	15 (27.3%)		28 (50.9%)	8 (14.5%)	5 (9.1%)		20 (36.4%)	35 (63.6%)	
>50	40 (76.9%)	0 (0.0%)	12 (23.1%)		32 (61.5%)	6 (11.5%)	3 (5.8%)		21 (40.4%)	31 (59.6%)	
Education Status											
Primary	17 (81.0%)	0 (0.0%)	4 (19.0%)	0.489	14 (66.7%)	1 (4.8%)	2 (9.5%)	0.678	7 (33.3%)	14 (66.7%)	0.149
Secondary	33 (82.5%)	1 (2.5%)	6 (15.0%)		24 (60.0%)	9 (22.5%)	2 (2.0%)		18 (45.0%)	22 (55.0%)	
Diploma	28 (73.7%)	0 (0.0%)	10 (26.3%)		20 (52.6%)	7 (18.4%)	2 (5.3%)		19 (50.0%)	19 (50.0%)	
University	64 (75.3%)	0 (0.0%)	21 (24.7%)		47 (55.3%)	11 (12.9%)	7 (8.2%)		26 (30.6%)	59 (69.4%)	

## Data Availability

The data used to support the findings of this study are available from the corresponding author upon request.
